# Glycation metabolites predict incident age-related comorbidities and mortality in older people with HIV

**DOI:** 10.1007/s11357-025-01652-3

**Published:** 2025-04-17

**Authors:** Xi Qiao, Liangliang Zhang, Emely A. Hoffman, Grace E. Mastin, Corrilynn O. Hileman, Asha R. Kallianpur, Ming Wang, Ronald J. Ellis, Susan L. Koletar, Frank J. Palella, Katherine K. Tassiopoulos, Alan L. Landay, Pankaj Kapahi, James J. Galligan, Robert C. Kalayjian, HAILO Study Consortium

**Affiliations:** 1 Department of Population and Quantitative Health Sciences, Case Western Reserve Univ, Cleveland, OH USA; 2 Case Comprehensive Cancer Center, Cleveland, OH USA; 3 Department of Pharmacology and Toxicology, University of Arizona, Tucson, AZ USA; 4 MetroHealth Medical Center, Cleveland, OH USA; 5 Case Western Reserve University School of Medicine, Cleveland, OH USA; 6 Cleveland Clinic Lerner Research Institute, Cleveland, OH USA; 7 Ohio State University, Columbus, OH USA; 8 Northwestern University Feinberg School of Medicine, Chicago, IL USA; 9 Harvard T.H. Chan School of Public Health, Boston, MA USA; 10 University of Texas Medical Branch, Galveston, TX USA; 11 The Buck Institute for Research on Aging, Novato, CA USA; 12 Louis Stokes Cleveland Veterans Affairs Medical Center, Cleveland, OH USA; 13 University of California San Diego, LaJolla, CA, USA

**Keywords:** Glycolysis, Advanced glycation end products, Methylglyoxal, Deoxyglucosone, Glyoxalase cycle, Glutathione, Lactoyl-glutathione, Lactoyl-lysine protein

## Abstract

Glycation is a class of modifications arising from non-enzymatic reactions of reducing sugars with proteins, lipids, and/or DNA, generating advanced glycation end-products (AGEs). AGEs are linked to many age-related comorbidities. In response to HIV-1 infection, activated T-cells and macrophages shift their predominate metabolism from oxidative phosphorylation to glycolysis. Increased glycolytic flux enhances AGE formation, which may increase age-related comorbidities. In this prospective, multicenter cohort study of antiretroviral therapy treated people with HIV, we explored predictive associations by baseline plasma AGE concentrations and their corresponding detoxification metabolites, with incident comorbidities and mortality. AGEs included dicarbonyl sugars: 3-deoxyglucosone, glyoxal, and methylglyoxal. Methylglyoxal-derived metabolites included carboxyethyl-arginine, carboxyethyl-lysine, and methylglyoxal hydroimidazolone-1. Detoxification metabolites included reduced and oxidized glutathione, and the glyoxalase cycle products lactoyl-glutathione and lactoyl-Lysine modified proteins. Plasma was collected at study entry, in the fasting state, and assayed by liquid chromatography-mass spectroscopy. Incident clinical outcomes included diabetes, chronic kidney disease, hypertension, neurocognitive impairment, peripheral neuropathy, frailty, fractures, recurrent falls, and all-cause mortality. Among 376 participants, higher baseline plasma concentrations of methylglyoxal derived AGEs predicted increased risks of diabetes, chronic kidney disease, and recurrent falls, while higher 3-deoxyglucosone predicted an increased risk of peripheral neuropathy. By contrast, higher baseline concentrations of reduced or oxidized glutathione, lactoyl-glutathione, and/or lactoyl-Lysine modified proteins predicted lower risks of diabetes, neurocognitive impairment, frailty, fractures, recurrent falls, and all-cause mortality. These findings support growing experimental evidence of the potential to mitigate age-related declines by interventions that reduce glycation or increase glutathione.

**Supplementary Information:**

The online version contains supplementary material available at 10.1007/s11357-025-01652-3.

## Introduction

Despite virally suppressive antiretroviral therapy (ART), people with HIV (PWH) have accentuated risks for age-related comorbidities, multimorbidity, and accelerated health declines that may occur at younger ages compared to appropriately matched people without HIV (PWoH) [1, 2]. Among the many possible mechanisms for this risk, PWH have heightened immune activation and oxidative stress with increased chronic inflammation compared to PWoH [3]. They also exhibit perturbed kynurenine and nicotinamide adenine dinucleotide (NAD+) metabolism, enhanced cellular senescence, and altered intestinal microbial metabolism [4–7].

HIV-1 infects activated CD4+ T-lymphocytes and induces the proliferation of classically activated, pro-inflammatory type 1 (M1) macrophages [8]. To meet the metabolic needs of rapid cellular proliferation, these activated T-cells and M1 macrophages shift their predominant metabolism from oxidative phosphorylation to aerobic glycolysis [9–12]. Ensuing increases in glycolytic flux promotes glycation, a non-enzymatic process by which reducing sugars covalently modify proteins, lipids, and/or DNA producing advanced glycation end products (AGEs) [13, 14].

AGEs circulate in plasma and bind to pattern recognition cell-surface receptors (receptors of AGE or RAGE) triggering proinflammatory and oxidative pathways [14–17]. AGEs also deposit in tissues including skin, adipose tissue, blood vessels, heart, kidneys, retina, brain, peripheral nerves, skeletal muscle, and bone, where they exert direct effects on the structure and function of their affected molecules [18–20]. They alter gene expression and metabolism and are linked to many age-related comorbidities including diabetes mellitus, kidney disease, atherosclerosis, heart failure, obesity, neurodegenerative disorders, bone fractures, and cancers [14, 18, 21–23].

**Fig. 1:**
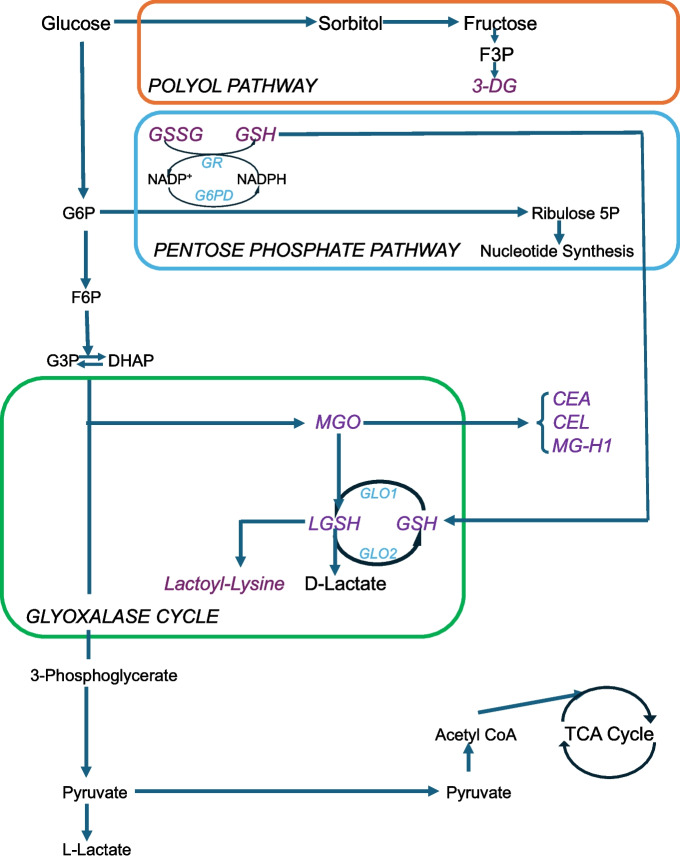
3-deoxyglucosone (3-DG) is generated from fructose in the polyol pathway. Methylglyoxal (MGO) is generated from the degradation of dihydroxy-acetone phosphate (DHAP) or glyceraldehyde- 3-phosphate (G3P) during glycolysis. Downstream metabolites of MGO include carboxyethyl-arginine (CEA), carboxyethyl-lysine (CEL), and methylglyoxal hydroimidazolone-1 (MG-H1). MGO is enzymatically detoxified to lactate in the glyoxalase cycle, wherein its lactate moiety is conjugated to glutathione via glyoxalase-1 (GLO1), forming lactoyl-glutathione (LGSH), which is then hydrolyzed to lactate via GLO2. With increased glycolytic flux, a rise in LGSH also promotes the transfer of this lactate moiety to form lactoyl-Lysine (lactoylLys)-modified proteins. Oxidized glutathione (GSSG) is reduced to GSH through the activity of glutathione reductase (GR), a NADPH-dependent enzyme

The most studied reactive sugars for AGE formation are the dicarbonyl glucose metabolites 3-deoxyglucosone (3-DG), glyoxal (GO), and methylglyoxal (MGO) [5–7, 9] (Fig. 1). Accumulating evidence implicates a central role of MGO derived metabolites in the pathogenesis of glycation related disease processes [18, 24, 25]. These include carboxyethyl-L-arginine (CEA), carboxyethyl-lysine (CEL), and methylglyoxal hydroimidazolone-1 (MG-H1), which circulate in plasma as free and protein bound adducts [14].

The glyoxalase cycle is a major detoxification system that converts MGO to lactate [26–28] (Fig. 1). Highly conserved in prokaryote and mammalian systems, glyoxalase-1(GLO1) conjugates the lactate moiety of MGO to reduced glutathione (GSH), yielding a lactoyl-glutathione (LGSH) intermediate [28]. LGSH then undergoes hydrolysis via GLO2, producing lactate while recycling GSH. With increased glycolytic flux, a rise in LGSH is observed, however, promoting the generation of lactoyl-Lysine (lactoylLys) modified proteins, where the lactate moiety from LGSH transfers instead to exposed lysine residues on these diverse proteins [26].

Limited data indicate that PWH have higher concentrations of circulating and skin AGE deposits compared to age and sex-matched PWoH [19]. We hypothesized that PWH have enhanced AGE formation that also contributes to an excess risk of age-related comorbidities. The objective of this study was to explore whether baseline plasma AGE concentrations, or their corresponding detoxification related metabolites, predict incident age-related comorbidities including diabetes mellitus, chronic kidney disease (CKD), fractures, recurrent falls, frailty, hypertension, peripheral neuropathy (PN), neurocognitive impairment (NCI), and all-cause mortality, each assessed among participants of the HIV Infection, Aging, and Immune Function Long-Term Observational Study (HAILO), a multicenter, prospective cohort study of older PWH.

## Methods

### Participants

HAILO is a multicenter, prospective, observational cohort study of PWH, ages 40 years or older at entry through the Advancing Clinical Therapeutics Globally (ACTG) network. Incident clinical outcomes for diabetes mellitus, CKD, hypertension, frailty, PN, and NCI, as defined and summarized in the Supplement (Table S1), were determined by chart review and/or direct measurements during follow-up through structured assessments that were conducted at entry, weeks 24, 48, and every 48 weeks thereafter. These assessments included questionnaires or surveys, clinical evaluations, functional measurements, neurological screening tests, and selected blood testing that were performed through the study or abstracted from the medical chart. Outcomes for fractures and recurrent falls were collected by self-report, and fractures were confirmed in the medical record. Mortality was assessed by notification from next-of-kin and confirmed in the medical record. For these analyses, baseline was defined as the HAILO entry visit. Participants were randomly selected from the entire HAILO cohort who had an available plasma specimen at entry and at least one post-entry follow-up assessment.

### Plasma metabolites

Plasma metabolite concentrations were assayed by liquid chromatography-mass spectrometry from frozen, stored specimens that were collected at study entry (baseline) in the fasting state (see also Supplemental Methods). These metabolites include the free carbonyl sugars: 3-DG, GO and MGO, and the free- and protein bound-MGO derived metabolites: CEA, CEL, and MG-H1 (Fig. 1). Detoxification-related metabolites included reduced and oxidized glutathione (GSH and GSSG, respectively), the glyoxalase cycle intermediates LGSH and lactoylLys modified proteins, as well as glucosyl-Lysine (glucosylLys) modified protein, wherein a GO adduct conjugates to exposed protein-Lysine residues of type I collagen [29].

### Statistical analysis

We conducted time-to-event analyses applying the Kaplan–Meier estimator to approximate survival probabilities for each of nine incident clinical outcomes. To examine the association between clinical outcomes, AGEs, and confounding variables, we adopted a two-step strategy. Potential confounders included age at study entry, sex assigned at birth (gender data was not available), race/ethnicity, duration of ART, education level, smoking history, nadir and entry CD4+ cell counts, HIV- 1 plasma viral load, plasma hemoglobin levels, and body mass index (BMI). Prior exposure to didanosine (ddI), stavudine (d4 T), or zalcitabine (ddC) was included in models of PN, and prior or current efavirenz exposure was included in models of NCI. First, we used univariate Cox proportional-hazards (PH) models, treating each incident outcome as the response variable and including each possible confounder, one at a time, as the covariate. This was followed by multivariable Cox PH models, where each time-to-event outcome was regressed on the AGE variable, one at a time, adjusting for the potential confounders and expressed as hazard ratios for each 1 S.D. increase in the plasma concentration of the metabolite of interest. The AGE variables were Box-Cox transformed to stabilize variance and approximate normal distributions using the R package *MASS*. Confounding variables for subsequent multivariable models were selected if they exhibited a *P*-value < 0.1. Significant confounding variables were identified by *P* < 0.1 in the multivariable models. *P*-values of AGE variable were adjusted using the Benjamini–Hochberg method at false discovery rate (FDR) of 0.05 for multiple testing correction. Chord diagrams, heatmaps, and survival continuous plots were drawn using the R package *circlize*, *pheatmap*, and *contsurvplot* to visualize and quantify the direction and magnitude of the associations between disease development and identified AGEs (*P* < 0.05). A detailed description of the statistical analysis methods is provided in the supplementary materials.

## Results

This analysis included 376 participants who were randomly selected among the entire cohort of 971 HAILO participants with an available plasma sample at entry. At baseline, the mean age was 51 years, 70 (19%) were female at birth, and 198 (52%) were of Black race or Hispanic ethnicity (Table 1). The mean duration of ART was 8.4 years, the mean baseline and nadir CD4+ cell counts were 652 and 214 cells/μL, respectively, and 319 (85%) had plasma HIV-1 RNA < 50 copies/mL. The mean follow-up was 4.3 years. There were no significant differences in any of the baseline variables between HAILO participants who were or were not included in this analysis (Table S2). Descriptive statistics of baseline glycation products and glutathione are summarized in Table 2. Among the plasma metabolites, only free-MG-H1 was weakly correlated with older age (*r* = 0.13, *P* = 0.01).

**Table 1:** Summary statistics of demographic and selected laboratory variables

Demographic variables	*N* = 376
Age^a^	51 ± 7 (40–77)
Sex^b^	
Female	70 (19%)
Male	306 (81%)
Race/ethnicity^b^	
Black, non-Hispanic	107 (28%)
Hispanic (regardless of race)	91 (24%)
White, non-Hispanic	178 (47%)
Body mass index^a^	27.8 ± 5.4 (18.4–52.2)
Formal education level (years)^a^	14 ± 4 (0–25)
≥ 12 years, *N* (%)^b^	305 (81%)
< 12 years, *N* (%)^b^	71 (19%)
Smoking status	
Current, *N* (%)^b^	94 (25%)
Never, *N* (%)^b^	150 (40%)
Past, *N* (%)^b^	132 (35%)
Duration of antiretroviral therapy (years)^a^	8.4± 4.1 (2.8 - 17.0)
Nadir CD4 + cell count (cells/μL)^a^	214 ± 166 (0–878)
Entry CD4 + cell count (cells/μL)^a^	652 ± 296 (45–2621)
Hemoglobin concentration (g/dL)^a^	14.38 ± 1.51 (8.70–17.70)
Missing^a^	12 (3%)
HIV RNA	
> 50 copies/mL^b^	57 (15%)
≤ 50 copies/mL^b^	319 (85%)
Prior exposure to efavirenz^b^	145 (39%)
Prior exposure to didanosine, stavudine or zalcitabine^b^	57 (15%)

^a^Mean ± S.D. (range), ^b^*N* (%)

**Table 2:** Plasma concentration of baseline AGEs and detoxification related metabolites. Baseline plasma metabolite concentrations derived from liquid chromatography-mass spectroscopy

**Metabolites of glycation**	**Mean (**± **SD), *N* = 376**
*Dicarbonyl-Sugars*	***μM***
MGO	5.05 ± 3.49 (0.94–40.60)
GO	0.98 ± 0.59 (0.20–7.31)
3-DG	0.12 ± 0.20 (0.01–3.05)
Missing *N*, %	1 (0.2%)
*MGO derived metabolites (free adducts)*	***μM***
*Free*-CEA	0.007 ± 0.006 (0.001–0.064)
*Free*-MG-H	0.76 ± 0.34 (0.22–3.16)
*Free*-CEL	0.044 ± 0.020 (0.006–0.143)
Missing *N*, %	2 (0.5%)
*MGO derived metabolites (protein bound)*	***pmole/nmole leucine***
*Protein bound*-CEA	1.45 ± 5.02 (− 0.11–97.90)
*Protein bound*-MG-H1	0.81 ± 1.50 (0.11–17.01)
*Protein bound*-CEL	0.03 ± 0.17 (− 0.12–2.98)
LactoylLys	0.04 ± 0.12 (− 0.06–1.80)
Missing *N*, %	1 (0.2%)
*GO derived metabolites*	
GlucosylLys	1.11 ± 1.57 (0.22–24.62)
*Glutathiones*	***nM***
GSH	6.18 ± 10.91 (0.78–150.0)
GSSG	0.06 ± 0.33 (0.01–5.30)
LGSH	3.26 ± 2.27 (0.25–19.03)
Missing *N*, %	4 (0.1%)

*3-DG*, 3-deoxyglucosone; *GO*, glyoxal; *MGO*, methylglyoxal; *CEA*, carboxyethyl-L-arginine; *CEL*, carboxyethyl-lysine; *MG-H1*, methylglyoxal hydroimidazolone; *GSH and GSSG*, reduced and oxidized glutathione, respectively; *LGSH*, lactoyl-glutathione; *lactoyLys*, lacotyl-Lysine modified proteins; *glucosylLys*, glucosyl Lysine; *μM*, micromolar; *nM*, nanomolar; *pmole*, picomole; *nmole*, nanomole

### Diabetes mellitus

**Fig. 2:**
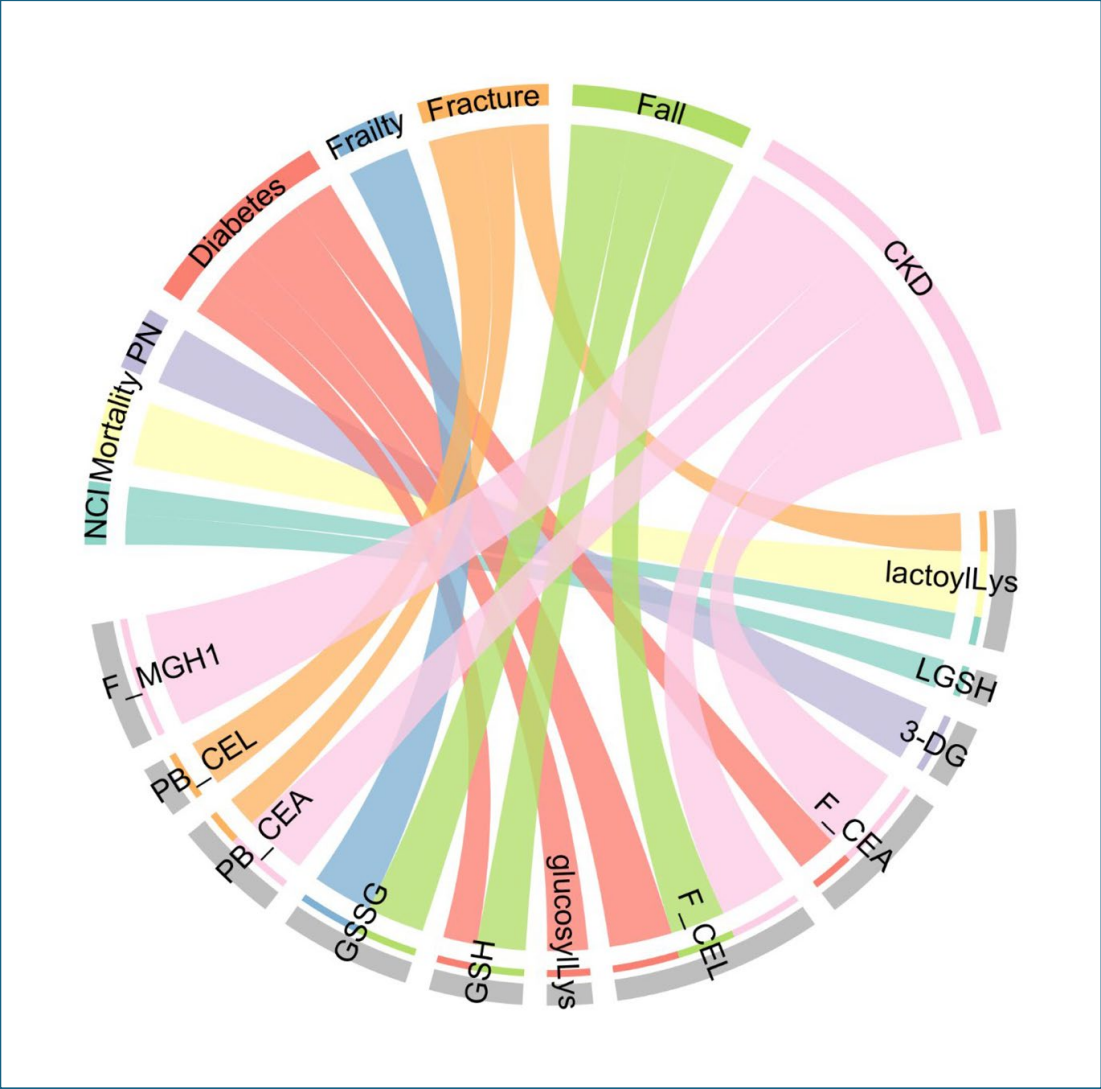
Chord diagram of the significant (*P* < 0.05) associations between time-to-event outcomes and AGEs in multivariable Cox proportional hazards models. The upper semicircle lists all time-to-event outcomes, which are distinguished by different colors, while the lower semicircle represents all AGE variables. “F_” and “B_” indicate free- and protein bound-metabolites, respectively. A connection between a time-to-event outcome and an AGE variable indicates a statistically significant association (*P* < 0.05) in the multivariable Cox PH models. The width of the band connection represents the magnitude of the estimated effect size

Fifteen participants (4.0%) had diabetes at entry, and an additional two lacked follow-up diabetes related data. Of the remaining 359 participants, 56 (15.6%) developed diabetes during follow-up (Fig. S1a). Higher BMI, longer ART exposure, lower plasma hemoglobin, non-smoking status, female sex at birth, and Black race or Hispanic ethnicity were associated with greater risks of incident diabetes (Fig. S1c). In multivariable models, higher baseline plasma concentrations of the circulating MGO derived metabolites free-CEA and free-CEL predicted a greater risk of incident diabetes (aHR = 1.36 [95% C.I. 1.02, 1.81], *P* = 0.04, and aHR = 1.62 [95% C.I. 1.22, 2.15], *P* < 0.01, respectively, for each 1 S.D. metabolite increase (Table 3; Figs. 2 & S1b)). Additional metabolic predictors of diabetes included higher plasma concentrations of glucosylLys modified protein (aHR = 1.37 [95% C.I. 1.04, 1.79], *P* = 0.03), and there was some evidence of an association of higher plasma 3-DG with this outcome (aHR = 1.25 [95% C.I. 0.98, 1.61], *P* = 0.08). Conversely, higher plasma GSH concentrations predicted a lower risk of incident diabetes (HR = 0.77 [95% C.I. 0.59, 0.997], *P* = 0.048).

**Table 3:** Results of multivariable Cox proportional hazard models of associations by AGEs and detoxification related metabolites with incident clinical outcomes. Exploratory metabolites are organized by the proximate dicarbonyl sugar from which they were derived

Dicarbonyl precursor or detox-metabolite	Incident outcome	AGE or detoxification-related metabolite	Estimate	Hazard ratio	SE	Test statistic^a^	*P*-value^a^	*P*-adjust^b^
3-DG	Peripheral neuropathy	3-DG	0.43	1.54	0.17	2.63	0.01	0.14
GO	Diabetes	GlucosylLys	0.31	1.37	0.14	2.25	0.02	0.19
MGO	Diabetes	*Free*-CEA	0.31	1.36	0.15	2.11	0.04	0.19
	Diabetes	*Free*-CEL	0.48	1.62	0.14	3.38	< 0.01	**0.01**
	Chronic kidney disease	*Free*-CEA	0.66	1.93	0.27	2.40	0.02	0.09
	Chronic kidney disease	*Free*-CEL	0.52	1.69	0.25	2.13	0.03	0.13
	Chronic kidney disease	*Protein bound*-CEA	0.48	1.62	0.19	2.56	0.01	0.08
	Chronic kidney disease	*Free*-MG-H1	0.88	2.42	0.27	3.30	< 0.01	**0.02**
	Recurrent falls	*Free*-CEL	0.42	1.52	0.17	2.42	0.02	0.15
	Fracture	*Protein bound*-CEA	− 0.24	0.79	0.11	− 2.25	0.02	0.13
	Fracture	*Protein bound*-CEL	− 0.26	0.70	0.10	− 3.50	< 0.01	**0.01**
GSH/GSSG	Diabetes	GSH	− 0.26	0.77	0.13	− 1.98	0.048	0.19
	Recurrent falls	GSH	− 0.37	0.69	0.18	− 2.13	0.03	0.18
	Recurrent falls	GSSG	− 0.44	0.64	0.19	− 2.34	0.02	0.15
	Frailty	GSSG	− 0.46	0.63	0.18	− 2.58	0.01	0.10
Glyoxalase cycle metabolite	Neurocognitive impairment	LGSH	− 0.22	0.80	0.10	− 2.23	0.03	0.20
	Neurocognitive impairment	LactoylLys	− 0.21	0.81	0.11	− 1.98	0.048	0.26
	Fracture	LactoylLys	− 0.29	0.75	0.12	− 2.41	0.02	0.13
	All-cause mortality	LactoylLys	− 0.48	0.62	0.21	− 2.23	0.03	0.41

^a^*P*-values were derived from the Wald test assuming the Chi-square distribution of the test statistics. ^b^*P*-values adjusted for multiple testing using Benjamini–Hochberg procedure. P-adjusted for correlates that remained signficant after adjusting for multiple testing are designated in **bold**. *3-DG*, 3-deoxyglucosone; *GO*, glyoxal; *MGO*, methylglyoxal; *CEA*, carboxyethyl-L-arginine; *CEL*, carboxyethyl-lysine; *MG-H1*, methylglyoxal hydroimidazolone; *GSH and GSSG*, reduced and oxidized glutathione, respectively; *LGSH*, lactoyl-glutathione; *lactoyLys*, lacotyl-Lysine modified proteins; *glucosylLys*, glucosyl Lysine; *μM*, micromolar; *nM*, nanomolar; *pmole*, picomole; *nmole*, nanomole

### CKD

Eighteen participants (4.8%) had CKD at entry, and there were 20 (6.0%) incident cases of CKD among the 330 participants with complete renal data. Older age predicted a greater risk of incident CKD (Fig. S1c). In the multivariable models, higher baseline concentrations of free- and protein bound-CEA (aHR = 1.93 [95% C.I. 1.13, 3.31], *P* = 0.02, and aHR = 1.62 [95% C.I. 1.12, 2.35], *P* = 0.01, respectively), free-CEL (aHR 1.69 [95% C.I. 1.04, 2.74], *P* = 0.033), and free-MG-H1 (aHR = 2.42 [95% C.I. 1.43, 4.09], *P* < 0.01) predicted greater risks of incident CKD (Table 3; Fig. 2).

### Hypertension

One hundred and forty-three participants (38.0%) had hypertension at entry. Of the remaining 231 participants with complete data, 20 (8.7%) developed hypertension during follow-up. Older age, higher BMI, and higher hemoglobin concentrations were associated with greater risks of incident hypertension (Fig. S1c). There were no significant associations between any glycation metabolite and incident hypertension in multivariable models.

### NCI

One hundred and one participants (26.9%) had NCI at entry, and 104 (40.0%) of the 267 participants with complete neurocognitive data developed NCI during follow-up. Higher BMI, lower nadir and baseline CD4+ cell counts, Hispanic ethnicity regardless of race, and less than 12 years of formal education were associated with greater risks of incident NCI (Fig. S1c). In multivariable models, higher baseline concentrations of the glyoxalase cycle metabolites LGSH and lactoylLys modified proteins predicted lower risks of incident NCI (aHR = 0.8 [95% C.I. 0.66, 0.97], *P* = 0.03, and 0.81 [95% C.I. 0.66, 0.99], *P* = 0.048, respectively; Fig. 2).

### PN

Twenty-three participants (6.1%) had Grade 1 or greater PN at entry. Among the 352 remaining participants with complete data, 43 (12.2%) developed PN during follow-up. Male sex, Hispanic ethnicity, lower nadir and baseline CD4+ cell counts, lower plasma hemoglobin concentrations, a history of current or past smoking, and prior exposure to ddl, d4 T, or ddC were associated with greater risks of incident PN (Fig. S1c). In multivariable models, higher baseline 3-DG plasma concentrations predicted an increased risk of incident PN (aHR = 1.54 [95% C.I. 1.12, 2.13], *P* = 0.01, Table 3; Fig. 2).

### Frailty

Eighteen participants (4.8%) were frail at entry, and 42 among the 342 participants with complete data (12.2%) developed frailty during follow-up. Older age, a history of past or current smoking, and less than 12 years of formal education were associated with greater risks of incident frailty (Fig. S1c). Higher baseline plasma GSSG concentrations predicted a lower risk of incident frailty (aHR = 0.63 [95% C.I. 0.45, 0.90], *P* = 0.01, Table 3; Fig. 2).

### Bone fracture

Thirty-three (8.8%) of 374 participants with complete fracture data experienced a fracture during follow-up. Older age, a current history of smoking, and higher baseline CD4+ cell counts were associated with higher risks of incident fracture (Fig. S1c). In multivariable models, higher baseline concentrations of protein bound-CEA and protein bound-CEL predicted lower risks of incident fracture (aHR = 0.79 [95% C.I. 0.64, 0.97], *P* = 0.02, and aHR = 0.70 [95% C.I. 0.57, 0.85], *P* < 0.001, respectively). Similarly, higher concentrations of lactoylLys modified proteins also predicted a lower risk of incident fracture (aHR = 0.75 [95% C.I. 0.59, 0.95], *P* = 0.02, Table 3; Fig. 2).

### Recurrent falls

Fifty-six (15.6%) of 359 participants with complete data regarding falls experienced 2 or more falls during follow-up. Older age, a history of current smoking, and less than 12 years of formal education were associated with greater risks of recurrent falls (Fig. S1c). In multivariable models, higher baseline concentrations of free*-*CEL predicted a greater risk of recurrent falls (aHR = 1.52 [95% C.I. 1.08, 2.15], *P* = 0.02). By contrast, higher baseline concentrations of both GSH and GSSG predicted lower risks of falls (aHR = 0.64 [95% C.I. 0.45, 0.93], *P* = 0.03, and aHR = 0.69 [95% C.I. 0.49, 0.97], *P* = 0.02, respectively; Table 3; Fig. 2).

### All-cause mortality

Ten (2.7%) participants died during follow-up. Lower entry CD4+ cell counts, lower plasma hemoglobin levels, and longer prior ART exposure predicted increased mortality (Fig. S1c). In multivariable models, higher baseline lactoylLys modified protein concentrations predicted a lower risk of death (aHR = 0.62, [95% C.I. 0.41, 0.94], P = 0.03, Table 3; Fig. 2).

## Discussion

Our study highlights the significant associations between metabolites of reactive dicarbonyl sugars and incident age-related comorbidities in older PWH. The detrimental effects of dicarbonyl stress from 3-DG, GO, and MGO derived metabolites were evident, corroborating with previous research addressing the impact of oxidative stress on cellular and tissue function [17, 30, 31]. The apparent protective associations by glutathione and the glyoxalase cycle metabolites LGSH and/or lactoylLys modified proteins mitigating these effects suggest potential therapeutic targets for reducing age-related health declines.

Accumulating experimental and clinical evidence among the general population suggests a central role of the dicarbonyl sugars 3-DG, GO, and MGO, and their respective AGEs, in the pathogenesis of age-related comorbidities [15, 23, 25, 32–35]. Consistent with this evidence, we also observed that higher baseline plasma concentrations of free or protein bound forms of the MGO derived metabolites CEA, CEL, and/or MG-H1 predicted greater risks of incident diabetes and CKD. We extend these associations by also demonstrating similar associations with recurrent falls. As in previous studies among the general population, we also observed that higher concentrations of glucosylLys modified protein predicted a greater risk of incident diabetes and found some evidence of such an association with 3-DG [29, 36, 37]. Furthermore, we detected a novel association of higher plasma 3-DG with a greater risk of PN. Unexpectedly, however, we observed protective associations by protein bound-CEA and -CEL with fractures, contrasting with findings from two previous studies among the general population that demonstrated a greater risk of incident hip fracture in association with higher antecedent concentrations of carboxymethyl-Lysine, a GO metabolite [23, 35].

GSH is an ubiquitous tripeptide that protects against free-radical induced oxidant injury [38]. The oxidized product GSSG is reduced to GSH through the activity of glutathione reductase, a NADPH-dependent enzyme (Fig. 1) [38]. Low plasma GSH concentrations were previously documented in older people and in PWH, diabetes, liver and neurodegenerative diseases, adult respiratory distress syndrome, and cystic fibrosis [39, 40]. Lower plasma GSH concentrations were also previously associated with increased mortality in PWH during the pre-ART era and predicted a greater risk of polymorbidity among older people in the general population [39, 41].

We observed protective associations by GSH and/or GSSG with incident diabetes, frailty, and recurrent falls that are consistent with the previously described antioxidant effects of glutathione in diabetes and with skeletal muscle physiology [42, 43]. In addition, we describe novel protective associations by LGSH and/or lactoylLys modified proteins with NCI, fracture, and all-cause mortality, implicating specific, beneficial effects by the glyoxalase cycle on these clinical outcomes. Previous studies in human cell culture systems demonstrated that glycolytic enzymes were overrepresented by lactoylLys modified proteins, and this modification was associated with reduced glycolysis [26]. The beneficial associations by lactoyLys modified proteins that we observed may therefore represent a negative feedback response to enhanced glycolysis from chronic HIV- 1 infection.

Experimental models provide accumulating evidence of pathology that is attributable to dicarbonyl stress and corresponding protective associations by the glyoxalase cycle enzyme GLO1. For example, in a transgenic diabetic rat model, overexpression of GLO1 prevented hyperglycemia induced MGO derived AGE formation in the neural retina, protecting it against capillary degenerative pathology [33, 44]. In a humanized HIV-1 mouse model, a two-fold increase in plasma MGO concentrations precipitated cardiac diastolic dysfunction in conjunction with histologic evidence of endothelial disruption and myocardial fibrosis [45]. Also in a mouse model, feeding a combination of dietary supplements to lower MGO derived glycation products (comprised of nicotinamide, pyridoxamine, thiamine, lipoic acid, and peperin) led to improved glucose homeostasis, better motor coordination and increased lifespan (manuscript submitted; 10.1101/2022.08.10.503411). In the present study, the beneficial associations with LGSH and lactoylLys modified proteins support the importance of the glyoxalase cycle in mitigating age-related health declines.

There are limited data addressing AGEs in PWH. In autopsied cardiac tissues from 7 deceased PWH and heart failure, MG-H1 tissue concentrations were significantly higher in vascular smooth muscle cells compared to samples from 14 deceased PWoH, with or without heart failure [45]. PWH had significantly higher concentrations of skin AGE deposits, determined by immunofluorescence, and higher plasma glyoxal hydroimidazolone 1 (GO-H1) concentrations than PWoH [19]. In a cross-sectional comparison of 68 PWH and 22 healthy PWoH, higher skin AGE concentrations were associated with increased arterial stiffness, while higher plasma GO-H1 concentrations were associated with heightened markers of oxidative stress, inflammation, and endothelial dysfunction, and these associations were evident only in the PWH [19]. In a study of 214 ART naïve participants who initiated ART through an ACTG clinical trial, MG-H1 serum concentrations increased significantly over the first 96 weeks of ART, and these increases were associated with insulin resistance [46]. Finally, in a single center cohort study of 91 antiretroviral therapy treated PWH, five of six participants who experienced an incident cardiovascular disease event had AGE skin concentrations that were above the study sample mean (*P* = 0.077 by log-rank) [47].

Reflecting the exploratory nature of this analysis, only three of the 20 significant associations that we detected remained significant (to *P* < 0.05) in multivariable models after adjusting for multiple testing. These include higher free-CEL plasma concentrations in association with incident diabetes (*P* = 0.01), higher free-MGH- 1 with incident CKD (*P* = 0.02), and higher protein bound-CEL with incident fracture (*P* = 0.01). As in previous studies among the general population, the consistent associations in the present study with diabetes by glucosylLys and 3-DG lend external validity to our findings [29, 36, 48]. We only examined associations by baseline metabolites with clinical outcomes. Future analyses using time-updated repeated measurements to examine the effects of changing metabolite concentrations on these clinical outcomes would be of interest.

## Conclusions

Higher baseline plasma concentrations of metabolites that are derived from the dicarbonyl reducing sugars 3-DG, GO, or MGO predicted higher risks of incident diabetes, CKD, recurrent falls, and PN in multivariable models. Protective associations were observed by GSH, GSSG, or the glyoxalase cycle metabolites LGSH and/or lactoylLys modified proteins with incident diabetes, NCI, frailty, fractures, recurrent falls, and all-cause mortality. These observations support growing experimental evidence of the importance of the glyoxalase cycle in age-related morbidities and the potential for interventions that reduce glycation and increase glutathione to mitigate age-related declines in PWH.

## Supplementary Information

Below is the link to the electronic supplementary material.

ESM 1 (DOCX 459 KB)

## Data Availability

Not applicable.
