# Supplementary material for: Glycation metabolites predict incident age-related comorbidities and mortality in older people with HIV
Source: GeroScience. 2025 Apr 17;48(1):379–90. doi: 10.1007/s11357-025-01652-3 (PMC12972458; doi:10.1007/s11357-025-01652-3)
Supplement: Supplementary file 1 — (DOCX 459 KB) [file 11357_2025_1652_MOESM1_ESM.docx]

**SUPPLEMENTAL INFORMATION**

**Glycation Metabolites Predict Incident Age-Related Comorbidities and Mortality in Older People with HIV**

**Supplemental Methods**

**Liquid chromatography-mass spectroscopy Assays of Plasma Metabolites**

**Statistical Analysis**

**Supplemental Tables**

**Table S1.** Detailed definitions of the clinical outcomes.

**Table S2.** Baseline variables between those who were or were not included in this analysis.

**Supplemental Figures**

**Figure S1 (a)** Kaplan-Meier survival curves showing the estimated survival probability by time for diabetes.

**Figure S1 (b)** Continuous survival plot illustrating the significant associations between diabetes and free-CEA (top left), free-CEL (top right), glucosylLys (bottom left), and GSH (bottom right). Blue and red represent the magnitude of the value of AGEs. Increased values of free-CEA, free-CEL, and glucosyl-Lysine are associated with a lower survival probability for diabetes, while increased values of GSH are associated with a higher probability of disease-free survival from developing diabetes.

**Figure S1 (c)** Heatmap of the estimated regression coefficients for significant (p<0.1) associations between time-to-event outcomes and confounding variables in the univariate Cox proportional hazards models. Red represents a positive association between the confounding variable and the hazard of developing the outcome (estimated hazard ratio >1), blue represents a negative association (estimated hazard ratio <1), and white represents a hazard ratio of 1. The color scale indicates the magnitude of the effect size.

**Supplement References**

**Supplemental Methods: Liquid Chromatography-Mass Spectroscopy Assays of Plasma Metabolites**

Ice-cold 80:20 MeOH:H 2 O (300 µL) was added to 10 µL serum and placed at -80ºC for & gt;1 h to precipitate protein. Samples were centrifuged (174,000 x g, 10 min, 4ºC) and the supernatant was split to three tubes (100 µL each) and assayed along with the pellet as below:

Free dicarbonyls: 50 pmol 13 C 3 -MGO and ο-phenylenediamine (50 µL, 1 mM final) was added to 100 µL supernatant and derivatized in the dark for 1 h. Samples were the clarified via centrifugation and 12 µL was chromatographed using a Shimadzu LC40 system equipped with a 50 × 2.1 mm, 3 μm particle diameter Atlantis C 18  column at a flow rate of 0.500 mL/min. Buffer A (0.1% formic acid (FA) in water) was held at 95% for 0.5 min then a linear gradient to 98% buffer B (0.1 % FA in ACN) was applied over the next 3 min. The column was held at 98% B for 2 min and then washed at 100% B for 0.5 min followed by re-equilibration at 95% A for 1.5 min. Multiple reaction monitoring (MRM) was conducted in positive mode using an AB SCIEX 6500 QTRAP+ with the following transitions: m/z 145.0 → 77.(MGO);  m/z 148.0 → 77.0 (13 C 3 -MGO); m/z 131.0 → 77.0 (GO); m/z 235.0 → 157.0 (3-DG). Samples were normalized to serum volume.

GSH, GSSG, and LGSH: To 100 µL of supernatant, added 150 µL 20% 5-sulfosalcylic acid (w/v) containing 2.5 1.25 nmol of GSH-(glycine- 13 C 2 , 15 N, internal standard). Samples were clarified via centrifugation and 12 µL was chromatographed using a Shimadzu LC system equipped with a 150 x3mm, 3 µm particle diameter Atlantis C 18 column (Waters, Milford, MA) at a flow rate of 0.400 mL/min. Solvent A (10 mM heptafluorobutyric acid (HFBA) in H 2 O) was held at 95% for 1 min, and lowered to 90% A over the next 9 min. Then, a linear gradient to 98% B (10 mM HFBA in ACN) was applied over the next 10 min. The column was held at 98% B for 4.5 min and then equilibrated to 95% A for 0.5 min. The needle was washed prior to each injection with a buffer consisting of 25 mM NH 4 OAc in MeOH for 2.5 min. MRM was performed in positive ion mode using an AB SCIEX 6500+ QTRAP with the following parameters: m/z 308.0 → 179.0 (GSH); m/z 311.0 → 182.0 (GSH-(glycine- 13 C 2 , 15 N)); m/z 613.0 → 355.0 (GSSG); m/z 380.1 → 233.1 (LGSH). Samples were normalized to serum volume.

Free MG-H1, CEA, CEL, glucosyl-Lys, lactoyl-Lys: To 100µL supernatant, 510 pmol 13 C-MG-H1, 510 pmol 13 C CEA and 1:1 heptafluorobutryic acid:H2O (105 µL, HFBA, 7.66 M) was added. Samples (12µL) were as chromatographed as described below.

Protein-bound PTMs: The serum protein pellet (above) was resuspended in 50 mM ammonium bicarbonate (65200 µL) and briefly sonicated. Samples were then spiked with internal standards and digested as described. Samples were chromatographed (12 μL) using a Shimadzu LC40 system equipped

**Supplemental Methods: Statistical Analysis**

Due to the longitudinal nature of outcomes such as diabetes, CKD, NCI, all-cause mortality, PN, hypertension, frailty, fracture, and recurrent falls in the HAILO study, we employed time-to-event analysis on these response variables in HIV-positive individuals aged 40 years and older who are on ART. The Kaplan-Meier estimator was used to approximate survival probabilities and assess the timing of disease development and progression, and Kaplan-Meier curve was drawn by using R package *survminer* [https://CRAN.R-project.org/package=survminer]. To understand the disease complexity with multiple outcomes, AGEs, and confounding variables, we adopted the following two-step analytical strategy.

In the initial step, to identify confounding variables associated with an increased or decreased hazard of developing specific disease outcomes for further investigation, we employed the following univariate Cox proportional-hazards (PH) models for each response variable against the confounding variables, one at a time:

$$\lambda\left( t | x_{i} \right)=\lambda_{0}\left( t \right)\exp(\beta_{x}X_{i}),$$

where $\lambda_{0}\left( t \right)$ stands for the baseline hazard at time point $t$, and $X_{i}$ represents the following confounding variable: age at study entry, sex assigned at birth, race/ethnicity, duration of prior ART, prior exposure to efavirenz, education level, smoking history, nadir CD4 count, CD4 count at entry, HIV viral load, plasma hemoglobin levels, and BMI. Missing values in confounding variables [Table 1] and AGEs [Table 2] were imputed using the R package *mice*, where each missing value was replaced with a random sample from the observed data. The continuous confounding variables were standardized, by subtracting from the study sample mean and dividing by the standard deviation. The estimations of the Cox PH model coefficients $\beta_{x}$ were obtained through the maximum likelihood estimation (MLE) method using the R package *survival* [https://CRAN.R-project.org/package=survival] . The estimated hazard ratio was computed by taking the exponential of estimated $\beta_{x}$. The p-values were derived from the Wald test assuming a Chi-square distribution of the test statistics. We adjusted the p-values using the Benjamini–Hochberg method to control the false discovery rate (FDR) associated with multiple testing (3). Significant confounding variables were identified by p < 0.1. Heatmaps were created using the R package *pheatmap* to better visualize the direction and magnitude of the associations between confounding variables and time-to-event outcomes.

Following this, to quantify the associations between AGEs while mitigating the influence of confounding variables, we employed multivariable Cox proportional hazards (PH) models. Each time-to-event outcome was considered as the response variable, and each AGE variable was treated as the exposure variable, one at a time, adjusting for the confounding variables to allow for more accurate assessments and interpretations of the associations between AGEs and outcomes:

$$\lambda\left( t | {A_{i}\boldsymbol{, C}}_{\boldsymbol{i}} \right)=\lambda_{0}\left( t \right)\exp(\beta_{A}A_{i}+\boldsymbol{\beta}_{\boldsymbol{C}}^{T}\boldsymbol{C\_i}),$$

where $A_{i}$ represents the AGE variable of interest, and $\boldsymbol{C}_{\boldsymbol{i}}\boldsymbol{=}\left( {C_{1}}_{i}\boldsymbol{, \ldots}{C_{j}}_{i}\boldsymbol{\ldots,}{C_{m}}_{i} \right)^{\boldsymbol{T}}$ is a $m$ by $1$ vector representing the $m$ potential confounding variables. The scaler $\beta_{A}$ and vector $\boldsymbol{\beta}_{\boldsymbol{C}}^{T}=\left( \beta_{C_{1}},\ldots\ldots,{\beta_{c}}_{M} \right)^{T}$ represent the associations between the hazard of disease development and AGE variables, as well as confounding variables, respectively. The AGE variables were Box-Cox transformed to stabilize the variance and approximate a normal distribution, with the power parameters $(\lambda)$ estimated using the MLE method by the R package *MASS*. The Box-Cox transformed AGE variables and continuous confounding variables were standardized. The $j$th confounding variables ${\boldsymbol{C}_{\boldsymbol{j}}}_{\boldsymbol{i}}$ were retained in the multivariable model if significant at p < 0.1. Significant AGEs were identified by p < 0.05. We employed a chord diagram using the R package *circlize* to visualize the overall associations between the set of identified AGEs and outcomes (4), and a heatmap to quantify the magnitude of these associations. To better illustrate how the survival probability of developing a specific disease outcome change over time in relation to increases and decreases in the identified AGE variable, we used continuous survival plots with the R package *contsurvplot,* with the covariate-level specific survival probabilities estimated using G-Computation (5). Finally, we adjusted the p-values of the AGE variables at FDR of 0.05.

**Table S1.** Detailed definitions of the clinical outcomes.

| **Incident Outcome** | **Definition** |
| --- | --- |
| Diabetes | Diabetes was defined by pharmacologic treatment for diabetes, or by a fasting glucose >126 mg/dL, a random plasma glucose >200 mg/dL, or a hemoglobin A1c > 6.5%, from plasma samples collected at entry and every 48 weeks during follow-up. |
| Chronic Kidney Disease (CKD) | CKD was defined using the creatinine-based, race neutral CKD epidemiology glomerular filtration (GFR) estimating equation, with two or more GFR measurements, separated by at least 3 months. |
| Neurocognitive Impairment (NCI) | NCI was assessed at entry and every 48 weeks using the NeuroScreen that included Trail Making A and B, Hopkins Verbal Learning, and the Wechsler Adult Intelligence Scale-Revised Digit Symbol test (1). Individual test scores for each domain were normalized and demographically adjusted. NCI was defined by at least 1 individual z-score ≥ 2 standard deviations (SD) below the mean, or at least 2 individual z-scores ≥ 1 SD below the mean. |
| Peripheral Neuropathy (PN) | PN was assessed at entry and every 48 weeks by trained non-neurologist site personnel. Grade 1 or greater neuropathy was defined as at least mild loss of vibration sensation in both great toes, or absent ankle reflexes bilaterally, or hypoactive ankle relative to the knee reflexes. |
| Hypertension | Hypertension was defined by antihypertensive treatment or a resting blood pressure ≥ 140/90 on ≥ 2 readings during separate visits. |
| Frailty | Frailty was assessed at entry and every 48 weeks using the Fried criteria (2). Weakness was assessed by the average of 3 dominant hand grip strength measurements using previous sex and body mass index (BMI) cutoffs. Slowness was defined as <1 m/sec from the average of 2 readings on a 4-meter walk. Weight loss was defined by self-report of ≥10 pounds unintentional weight loss during the past year. Low activity was defined as being “limited a lot” in response to “Does your health limit you in vigorous activities such as running, lifting heavy objects, or participating in strenuous sports?”. Exhaustion was defined as experiencing the feeling that “everything I do is an effort” or “sometimes I just cannot get going” at least 3–4 times per week. Participants were considered non frail if they met 0 components, prefrail if they met 1 or 2 components, and frail if they met 3–5 components. |
| Fractures | Fractures as detected by X ray, by self-report for any such event that occurred since the previous study visit. |
| Recurrent Falls | Recurrent Falls were determined by self-report consisting of at least 2 falls after enrollment. A fall was defined as an unexpected event in which the individual loses their balance and lands on the floor, ground or a lower level or hits an object. Events resulting from a major medical event such as a stroke or an overwhelming external hazard such as being pushed are not considered falls. |

**Table S2.** Baseline variables between those who were or were not included in this analysis.

| **Characteristic** | **N** | **Selected**  N = 376 | **Not Selected**  N = 595 | **P-value**^3^ |
| --- | --- | --- | --- | --- |
| **Age**^1^ | 971 | 51.47 (7.21) | 51.64 (7.73) | 0.73 |
| **Sex** | 971 |  |  | 0.70 |
| Female^2^ |  | 70 / 376 (19%) | 118 / 595 (20%) |  |
| Male^2^ |  | 306 / 376 (81%) | 477 / 595 (80%) |  |
| **Race** | 971 |  |  | 0.09 |
| Black, Non-Hispanic^2^ |  | 107 / 376 (28%) | 184 / 595 (31%) |  |
| Hispanic (Regardless of Race)^2^ |  | 91 / 376 (24%) | 109 / 595 (18%) |  |
| White, Non-Hispanic^2^ |  | 178 / 376 (47%) | 302 / 595 (51%) |  |
| **Duration of antiretroviral therapy**^2^ | 971 | 8.44 (4.06) | 8.48 (4.11) | 0.88 |
| **Education level (years)**^2^ | 971 | 13.59 (3.65) | 13.64 (3.59) | 0.85 |
| **Entry Body Mass Index**^1^ | 969 | 27.83 (5.35) | 28.18 (5.62) | 0.34 |
| Missing^4^ |  | 0 (0%) | 2 (0.3%) |  |
| **Entry Hemoglobin**^1^ | 947 | 14.38 (1.51) | 14.32 (1.58) | 0.58 |
| Missing^4^ |  | 12 (3.2%) | 12 (2.0%) |  |
| **Nadir CD4 cells (per μL)**^1^ | 971 | 214.37 (165.95) | 200.64 (160.44) | 0.20 |
| **Entry CD4**^1^**(per μL)** | 971 | 651.80 (295.83) | 664.21 (314.42) | 0.53 |
| **Entry HIV viral load (Log_10_)**^1^ | 971 | 1.70 (0.52) | 1.72 (0.50) | 0.58 |
| **Entry Smoking status** | 968 |  |  | 0.69 |
| Never^2^ |  | 150 / 376 (40%) | 247 / 592 (42%) |  |
| Past^2^ |  | 132 / 376 (35%) | 192 / 592 (32%) |  |
| Current smoker^2^ |  | 94 / 376 (25%) | 153 / 592 (26%) |  |
| Missing^4^ |  | 0 (0%) | 3 (0.5%) |  |
| **Follow-up (weeks)**^1^ | 971 | 226.36 (55.51) | 221.99 (52.47) | 0.22 |
| ^1^Mean (S.D.); ^2^n / N (%). ^3^Two Sample t-test for continuous variables; Pearson's Chi-squared test for categorical variables.^4^Number of missing values; No.(%) | | | | |

**Supplemental Figures**

**Figure S1 (a):** Kaplan-Meier survival curves showing the estimated survival probability by time for diabetes.

**Figure S1(b):** Continuous survival plot illustrating the significant associations between diabetes and free-CEA (top left), free-CEL (top right), glucosylLys (bottom left), and GSH (bottom right). Blue and red represent the magnitude of the value of AGEs. Increased values of free-CEA, free-CEL, and glucosyl-Lysine are associated with a lower survival probability for diabetes, while increased values of GSH are associated with a higher probability of disease-free survival from developing diabetes.

**Fig S1 (c):** Heatmap of the estimated regression coefficients for significant (p<0.1) associations between time-to-event outcomes and confounding variables in the univariate Cox proportional hazards models. Red represents a positive association between the confounding variable and the hazard of developing the outcome (estimated hazard ratio >1), blue represents a negative association (estimated hazard ratio <1), and white represents a hazard ratio of 1. The color scale indicates the magnitude of the effect size.

**Supplement References**

1. Ellis RJ, Evans SR, Clifford DB, Moo LR, McArthur JC, Collier AC, et al. Clinical validation of the NeuroScreen. J Neurovirol. 2005;11(6):503-11.

2. Erlandson KM, Wu K, Koletar SL, Kalayjian RC, Ellis RJ, Taiwo B, et al. Association Between Frailty and Components of the Frailty Phenotype With Modifiable Risk Factors and Antiretroviral Therapy. J Infect Dis. 2017;215(6):933-7.

3. Benjamini Y, Drai D, Elmer G, Kafkafi N, Golani I**.** Controlling the false discovery rate in behavior genetics research. Behav Brain Res. 2001;125(1-2):279-84.

4. Gu Z, Gu L, Eils R, Schlesner M, Brors B**.** circlize Implements and enhances circular visualization in R. Bioinformatics. 2014;30(19):2811-2.

5. Denz R, Timmesfeld N**.** Visualizing the (Causal) Effect of a Continuous Variable on a Time-To-Event Outcome. Epidemiology. 2023;34(5):652-60.
